# Look granulomatosis with polyangiitis (GPA) straight in the face: missed opportunities leading to a delayed diagnosis

**DOI:** 10.1186/s13317-019-0118-4

**Published:** 2019-09-17

**Authors:** N. Rolle, M. Muruganandam, I. Jan, F. M. Harji, J. Harrington, K. N. Konstantinov

**Affiliations:** 1 Department of Medicine, Division of Rheumatology, University of New Mexico School of Medicine, Albuquerque, NM 87131 USA; 2 Section of Rheumatology, Raymond G. Murphy Veterans Affairs Medical Center, 1501 San Pedro SE, Albuquerque, NM 87108 USA

**Keywords:** Granulomatosis with polyangiitis (GPA), Bilateral facial palsy, Aortic mural thrombus

## Abstract

Granulomatosis with polyangiitis (GPA) is a systemic vasculitis with a potential to involve any organ system. It remains an important cause of kidney related morbidity and mortality. Early diagnosis can be difficult and requires high index of suspicion in all patients, but especially in cases with atypical presentation. We report a case with GPA, which was diagnosed only after new and advancing symptoms belied the original diagnosis of bilateral facial palsy and aortic mural thrombus.

## Introduction

Granulomatosis with polyangiitis (GPA) is a rare disease with annual incidence of 5 to 10 cases per million [1]. The initial phenotypic expression of GPA exhibits considerable variability, which often goes beyond the site of onset, degrees of involvement of different systems, and rate of progression. GPA is labeled as one of the great masqueraders in medicine, because it may present with protean symptoms requiring a broad differential diagnosis. The pre-diagnostic period of GPA may last from a few months to many years [2]. Due to involvement of multiple organ systems, it is not uncommon for patients to seek help from a variety of clinician specialists [3]. Making the correct diagnosis is even more challenging when the presenting symptoms may not fit the classic picture of the disease. In such cases, the diagnostic delay increases the risk of disease progression, organ failure, or even death.

We report a case of GPA in a 65-year-old immunocompetent man, who presented with progressive bilateral facial palsy and a mural thrombus in the thoracic and abdominal aorta. Initially, these clinical manifestations could not be attributed to any particular etiology. This unusual clinical presentation illustrates the need for increased awareness of vasculitis for early diagnosis and timely treatment of this life-threatening condition.

## Case report

A 67-year-old man with uncontrolled diabetes mellitus, peripheral neuropathy, and 40 lbs. unintentional weight loss within the past year presented to an outside hospital with 2 days history of left facial weakness, flattening of the left nasolabial fold, and inability to close his left eyelid. He was afebrile, hemodynamically stable, without systemic complaints or other pertinent findings. Routine laboratory data were unremarkable, including urinalysis, red and white blood cell counts, basic metabolic panel, and chest X-ray. Magnetic resonance imaging (MRI) and computed tomography scans (CT) of the brain were unremarkable. He was diagnosed with Bell’s palsy and discharged on prednisone 60 mg/day for 7 days and empiric Acyclovir.

The patient’s symptoms continued to worsen progressively despite the steroid treatment. Two weeks later he was admitted with bilateral facial and eyelid weakness. He had difficulty swallowing and was unable to take his oral medications for the last several days. He denied fever, chills, cough, hemoptysis, epistaxis, hearing impairment, headache, chest pain and palpitations, dysuria, hematuria, rash, and joint swelling. His family history was notable for a mother who died of throat cancer and a father who died from leukemia.

**Fig. 1:**
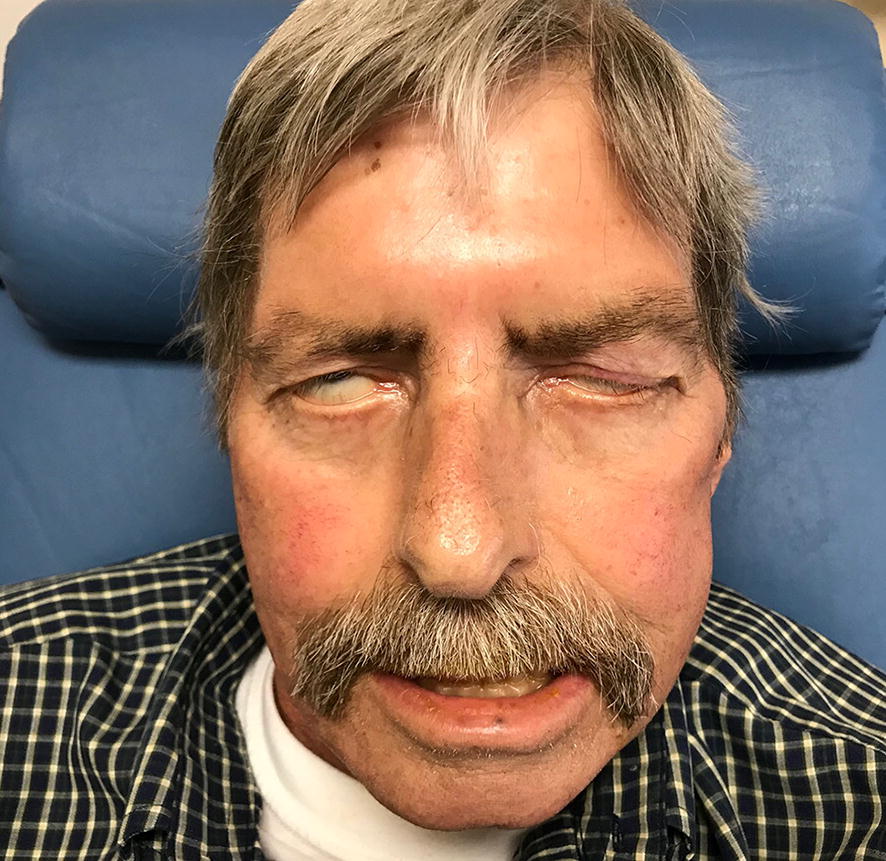
Granulomatosis with polyangiitis: isolated bilateral facial nerve palsy at presentation, which developed into systemic disease three months later with new-onset renal failure

**Fig. 2:**
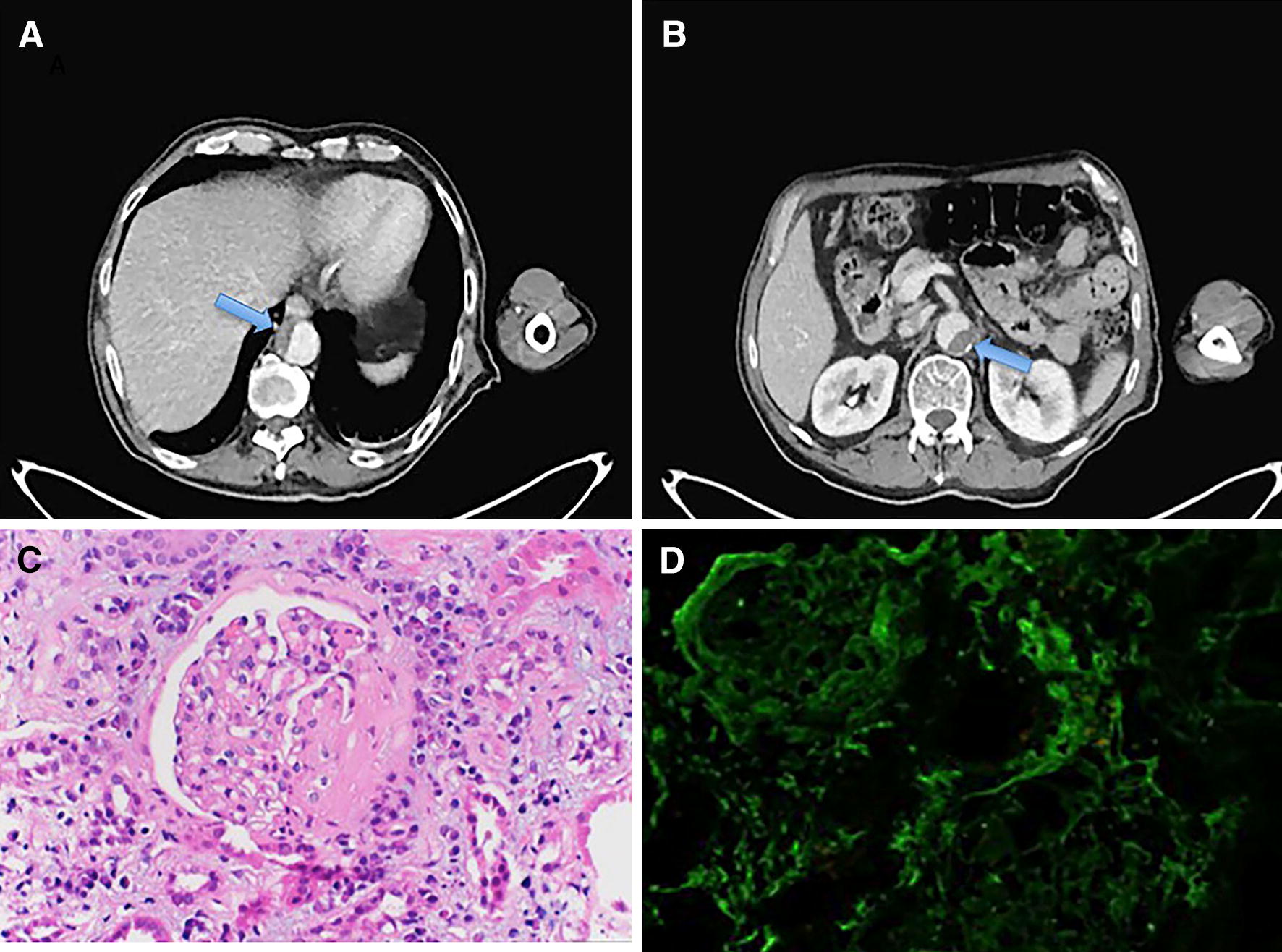
Full body positron emission tomography (PET) CT showing thoracic aorta mural thrombus (**A**) and abdominal aorta mural thrombus (**B**). Renal biopsy showing segmental necrotizing glomerulonephritis with crescentic features on light microscopy (**C**) and localization of fibrinogen and absence of immune-complex type deposits on immunofluorescence; pauci-immune glomerulonephritis (**D**)

On examination, he was afebrile with normal heart rate and blood pressure, but was tachypneic at 32 breaths per minute. He was pale with marked temporal wasting, had a scaphoid abdomen and bilateral pedal edema. Neurologically, he had findings consistent with bilateral 7th nerve palsy (Fig. 1), was unable to close his eyes and had excessive lacrimation as well as difficulty swallowing. Otoscopic examination did not show any abnormality. No skin rash, lymphadenopathy, or synovitis were noted. Laboratory data revealed neutrophilia of 11.4 k/mm^3^ (normal range 1.8–7) with a white cell count (WBC) of 16.3 k/mm^3^, serum creatinine of 0.92 mg/dL, estimated glomerular filtration rate 82 mL/min and normal urinalysis. Erythrocyte sedimentation rate (ESR), C-reactive protein (CRP) and rheumatoid factor (RF) were all mildly elevated. Multiple studies including brain MRI, Lyme disease serology, antinuclear antibodies (ANA), HIV, HSV type 1 and 2, EBV-specific IgM, acetylcholine receptor (AChR) antibody, muscle specific kinase (MuSK) antibody, Hep C antibody, Treponema pallidum RPR with reflex to titer, angiotensin converting enzyme (ACE), aldolase, TSH, and B12 were all negative or normal. Full body positron emission tomography (PET) CT showed segmental mural thrombus (Fig. 2a, b) involving the descending thoracic and abdominal aorta with up to 50% narrowing, but no aortic aneurysm or any neoplasia. Antiphospholipid antibodies, and protein C and S plasma levels were normal. Transthoracic ECHO-cardiogram showed no cardiac pathology. He was started on aspirin, atorvastatin, and clopidogrel. Based on collected data and symptoms he was clinically diagnosed with bilateral Bell’s palsy and discharged.

A month later the patient was seen in his primary care’s office with continued inability to close his eyelids. He was prescribed lubricant eye drops along with warm compresses to the eyelids.

Six weeks later, he was admitted again with additional weight loss, dysphagia, foul smelling urine, and progressive facial palsy. He endorsed chills for several days, but denied dysuria, or abdominal pain. Laboratory investigation revealed leukocytosis, mild anemia, serum creatinine of 6.8 mg/dL, lactic acid of 3.7 mmol/L, and serum glucose of 242 mg/dL. Arterial blood gases showed respiratory alkalosis and urinalysis was significant for hematuria, proteinuria, and numerous white blood cells. The working diagnosis at this time included sepsis due to urinary tract infection with acute kidney injury. Initially, the renal function of the patient improved slightly with intravenous fluid replacement, indwelling urinary bladder catheter insertion, and Ceftriaxone 1 gr/iv/daily. However, his serum creatinine continued an overall upward trend subsequently. Urine and blood cultures were negative. The differential diagnosis was expanded to include vasculitis and lupus nephritis. CT of neck and soft tissues found no evidence of subglottic airway narrowing or paranasal sinuses opacification (not shown). A percutaneous renal biopsy was performed. On light microscopy, glomeruli showed glomerular basement membrane (GBM) breaks, necrosis, and crescents at various stages of organization, moderate interstitial fibrosis and lymphocytic infiltrates within expanded interstitial spaces. Red cells and red cell casts were identified in the lumen of tubules (Fig. 2c). Immunofluorescent histology revealed a paucity of immunoglobulin deposits, negative C1q, and some fibrinogen irregular capillary wall staining (Fig. 2d). Assays for antineutrophil cytoplasmic antibodies (ANCA) revealed positive cytoplasmic staining pattern (c-ANCA), and positive anti-proteinase 3 antibodies (PR3-ANCA). The combination of pauci-immune crescentic glomerulonephritis and serum PR3-ANCA positivity confirmed the diagnosis of GPA. He received intravenous methylprednisone, 1 gm. daily for 3 days, and then transitioned to oral prednisone 1 mg/kg daily. He was started on cyclophosphamide 500 mg/m^2^ with renal adjustment once a month for 6 months. Atovaquone was started for Pneumocystis jiroveci pneumonia (PJP) prophylaxis. 4 months after initiation of immunosuppressive therapy the patient required hemodialysis due to end-stage renal disease. He showed improved facial impairment and reported no functional limitations from it.

## Discussion

GPA is a rare ANCA-associated multisystem necrotizing vasculitis that causes irreversible damage to affected organs, especially the respiratory tract and kidneys [3]. In substantial percent of GPA patients, the diagnostic uncertainty at disease onset reflects the clinical complexity and protean manifestations of the disease, the low-positive predictive value of the symptoms which are non-specific, and the high frequency of co-morbidities. Several studies have reported diagnostic delays that vary from a few months to several years [4, 5]. This case illustrates the challenges of a prompt GPA diagnosis in a patient with rare alarm symptoms, different from the more usual “red flags” associated with this disease (nasal ulceration with discharge, recurrent sinusitis, serous otitis media, stridor due to subglottic stenosis, pulmonary-renal syndrome). Post-hoc judgment in our case suggests that alternative approaches could have led to timely diagnosis.

The presenting symptom in our patient was a bilateral facial palsy. By itself, this is an extremely rare finding [6] which unlike the unilateral presentation is seldom secondary to Bell’s palsy. It occurs in 1 per 5,000,000 population and warrants a diagnostic work up because most of its causes are systemic diseases, some of which are life-threatening [7]. Differential diagnosis of such cases is challenging as it encompasses infectious, autoimmune, neoplastic, metabolic, toxic, and idiopathic factors. The majority of patients with bilateral facial palsy have sarcoidosis, Lyme disease, Guillain-Barré syndrome, tumor, or meningitis [8]. While the causal mechanisms for most of the cases with facial palsy remain elusive, the autoimmune pathogenesis of GPA has been also added to the list. The serologic hallmark of GPA is the presence of anti-neutrophil cytoplasmic autoantibodies (ANCA) predominantly directed against the proteinase 3 (PR3) and myeloperoxidase (MPO) enzymes released by neutrophils and monocytes upon cell activation [9]. In the absence of systemic signs of vasculitis at onset, only ANCA positivity could have allowed us to make the diagnosis of GPA.

During the initial diagnostic work-up, a hybrid PET/CT imaging was used to evaluate the patient for infectious, inflammatory, or neoplastic processes. Interestingly, it identified instead asymptomatic aortic mural thrombus (AMT) that had developed in both thoracic and abdominal aorta in the absence of known atherosclerotic or aneurysmal disease. Following the initial description by Weismann and Tobin [10]  in 1958, AMT has been accepted as a definite clinical entity and a source of arterial thromboembolism. AMT is very rare in the absence of aortic disease, since the aortic blood flow is too fast for clotting and forming of a growing thrombus. The thrombus is usually located in the abdominal aorta, and most commonly occurs in young women with a history of smoking.

In the majority of published cases with AMT, there was a previously undiagnosed hypercoagulable state associated with the presence of malignancies, blood clotting disorders, infectious or genetic disorders of the aortic wall [11]. Diabetes is known to predispose to cardiovascular disease, which was not evident in our patient. It is also intriguing that studies so far suggest a protective role of diabetes on the development of abdominal aortic aneurysm (AAA), often seen with AMT. In recent years an increased frequency of both venous and arterial events in ANCA-associated vasculitis has been reported in the literature [12]. These events are seen as a typical complication of GPA, especially in active disease with PR3-positivity [13]. Our patient had been tested for antiphospholipid antibodies, protein C and S, but not for ANCA that could have guided diagnosis and clinical decisions earlier.

Marked constitutional symptoms such as fever, weight loss, and fatigue add another layer of GPA complexity, ultimately resulting in a delayed diagnosis. The pattern of symptom progression ranges from mild and gradual to rapid life-threatening organ failure [14]. The progression of clinical manifestations in our case was initially attributed to metabolic or infectious causes.

## Conclusion

GPA is a rare entity with a high risk for life-threatening complications. Early recognition of the disease and matching a provisional with the final diagnosis is important for timely treatment and prevention of progression. This case illustrates the fact that bilateral facial palsy and AMT, both of which are rare in this clinical setting, can contribute to missed opportunities to diagnose GPA. In cases presenting with similar highly atypical symptoms and signs, the clinician should suspect systemic vasculitis first and then establish it by serology and tissue biopsy. We advocate an early consideration of ANCA testing beyond the pretest probability challenge to reach prompt diagnosis.

In 2013 the ACR Choosing Wisely campaign developed a list of screening and diagnostic tests thought to be over utilized [15]. While the resulting list did not include ANCA, this case supports the suggestion that the goal of immunologic testing need to be reconsidered and expanded beyond the goal to confirm a diagnosis with intention to treat, to a goal of case finding of pre-or early disease with an intent to prevent disease and end-organ damage [16].

## Data Availability

All patient’s data is part of the computerized patient record system (CPRS) of the Department of Veteran Affairs.
